# Fatty changes associated with N-Nitrosodiethylamine (DEN) induced hepatocellular carcinoma: a role of sonic hedgehog signaling pathway

**DOI:** 10.18632/genesandcancer.203

**Published:** 2020

**Authors:** Anindita Tripathy, Sudhir Thakurela, Manoj Kumar Sahu, Kanishka Uthansingh, Ayaskanta Singh, Jimmy Narayan, Amrendra Kumar Ajay, Vinay Singh, Ratna Kumari

**Affiliations:** ^1^ Disease Biology Laboratory, KIIT School of Biotechnology, KIIT University, Bhubaneswar, India; ^2^ Broad Institute of MIT and Harvard, Cambridge, MA, USA; ^3^ Department of Stem Cell and Regenerative Biology, Harvard University, Cambridge, MA, USA; ^4^ Department of Gastroenterology and Hepatobiliary Sciences, IMS and SUM Hospital, Bhubaneswar, India; ^5^ Brigham and Women’s Hospital, Harvard Medical School, Boston, MA, USA

**Keywords:** hepatocellular carcinoma, adiposity, SHH-E2F1 pathway, lipogenic molecules, adiponectin

## Abstract

Backgrounds and Aims: Hepatocellular Carcinoma (HCC) is the leading cause of cancer-related mortality across the world. Non-viral etiological factors including obesity and metabolic syndrome have now become prevalent cause of hepatocellular carcinoma. Sonic Hedgehog (SHH) pathway is activated in hepatocellular carcinoma but its role in regulation of lipogenic molecules during the hepatocarcinogenesis is not known. The aim of present study is to explore the role of SHH pathway in fatty changes associated with hepatocarcinogenesis at different stages and to further correlate the expression of SHH with lipogenic pathways.

Results: Our results demonstrated significant increase in lipidosis and fibrosis in DEN+CCl4 treated animals. It was simultaneously associated with the enhanced expression level of SHH, E2F1, adiponectin, and lipogenic molecules in DEN+CCl4 treated animals. These results were also corroborated with the similar findings in higher stage patients’ biospecimens.

Methods: N-Nitrosodiethylamine (DEN) and Carbon TetraChloride (CCl4) induced hepatocellular acrcinoma model in male Wistar rats were established to study the expression level of SHH pathway and associated fatty changes during different stages of hepatocarcinogenesis. The expression levels of SHH, E2F1, and lipogenic molecules were checked at different stages of hepatocellular carcinoma. These results were further compared with biospecimens of hepatocellular carcinoma patients of different stages.

Conclusions: Our results revealed an unknown aspect of SHH pathway in hepatocarcinogenesis via its control over lipogenesis. It gives insight into the lipogenic properties of DEN+CCl4 induced rodent hepatocarcinogenesis model and how SHH pathway operate to arbitrate this response.

## INTRODUCTION

Hepatocellular Carcinoma (HCC) is the most common primary liver cancer and the third leading cause of cancer-related death worldwide. In most cases *hepatocellular carcinoma* is refractory to the available chemotherapeutic drugs [1, 2]. The etiology of *hepatocellular carcinoma* is diverse including viral infections (HBV and HCV), metabolic syndrome, alcohol consumption, aflatoxin exposure, and hereditary factor (alpha-1 antitrypsin deficiency). Metabolic Syndrome (MetS) is a group of metabolic factor abnormalities (biochemical and physiological) associated with the global epidemic diseases like obesity, diabetes, and cardiovascular disease [3]. *Metabolic syndrome* is now considered a well documented risk-factor for Non-alcoholic Fatty Liver Diseases (NAFLD), which is a metabolic liver disease and can in turn lead to Non-Alcoholic Steatohepatitis (NASH) and fibrosis. Furthermore, fibrosis can lead to cirrhosis which subsequently can progress into hepatocellular carcinoma.

In order to drive carcinogenesis the metabolic pathways are rewired in cancer cells which supports their increased demand for metabolites and energy. Usually the normal cells take up exogenous fatty acids for lipid biosynthesis, but cancer cells are diverted towards *de novo* lipid biosynthetic pathway despite abundance of exogenous lipids. Nowadays, this particular metabolic shift is considered as one of the hallmarks of cancer [5]. There is now enough evidence which suggests that enhanced *de novo* lipid biosynthesis is a significant feature of several types of cancer [5]. Since the worldwide prevalence of obesity and other *metabolic syndrome* has increased enormously in last few decades, consequently the incidence of non-viral *hepatocellular carcinoma* has also increased. The deposition of adipose tissue in obese individuals is heterogeneous and adiposity of abdominal compartment mainly the visceral one is associated with majority of obesity associated pathologies [6]. Accumulation of visceral adipose tissue is accompanied with the proinflammatory cytokine and adipokine production and is associated with increased malignancy risk of various organs [7-10]. Moreover, visceral adiposity has been demonstrated to be an independent risk-factor for HCC recurrence after curative treatment [11].

N-Nitrosodiethylamine (DEN) is well known environmental hepatocarcinogen and it has been characterized as group I human carcinogen by World Health Organization [12]. DEN induced rodent hepatocarcinogenesis model has been successfully used to study impact of several drug treatment on hepatocellular carcinoma [13] and also shows histopathological similarities to human hepatocellular carcinoma [14]. Fatty metamorphoses is a well known phenomena during the hepatocarcinogenesis of humans [15] and several investigators have shown the use of DEN and high fat diet to induce Non Alcoholic Fatty Liver Disease related symptoms [16]. Chen et al., (2011) demonstrated the occurrence of fatty metamorphoses after DEN treatment in Syrian golden hamster model of hepatocarcinogenesis [17], but the molecular association between fatty metamorphoses and hepatic carcinogenesis is not clear up till now. We have already published our study demonstrating comprehensive change in Wnt and Hedgehog (Hh) signaling pathways in DEN + CCl4 induced rodent hepatocellular carcinoma model at different stages of hepatocarcinogenesis [18]. In the present study we identified the role of Sonic Hedgehog (SHH) pathway in fatty changes associated with DEN + CCl4 induced hepatocellular carcinoma model at different stages and substantiated the findings with clinical-samples. Indeed we correlated the change in fat accumulation in and around the liver of animals after DEN + CCl4 treatment with the simultaneous change in the levels of SHH.

## RESULTS

### DEN + CCl4 induced hepatocarcinogenesis was associated with visceral adiposity

**Figure 1:**
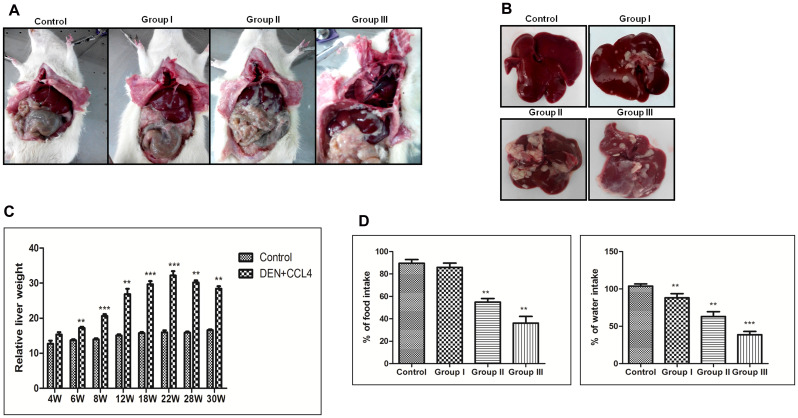
DEN+CCl4 induced hepatocarcinogenesis was associated with visceral adiposity. **A**. Fat accumulation in DEN + CCL4 treated rats along with Control. **B**. Increasing hepatic foci/ nodules observed in DEN + CCl4 treated rats. Rats were sacrificed at different stages of hepatocarcinogenesis. **C**. Measurement of liver weight showing increasing trend in the DEN + CCL4 treated groups. **D**. Decreasing food and water intake in treated groups. Data represented are representative of three independent experiments performed in triplicates and expressed as Mean ± SE, **p < 0.001, ***p < 0.0001.

The DEN + CCl4 model of male Wistar rat hepatocarcinogenesis was followed in our experiment [18]. We observed altered hepatic foci in treated animals at completion of the 8^th^week treatments followed by latency-period of two weeks. It was simultaneously associated with visual fat accumulation in and around the liver. There was significant accumulation of abdominal adipose tissue particularly in the visceral compartment and it was more in the group II animals (Figure 1A), whereas there was no such visible adiposity in control animals. The size and number of nodules was also more in group II animals (Figure 1B). The relative liver weight was increased in DEN + CCl4 treated group (Figure 1C) and there was a significant decrease in food and water intake of this group (Figure 1D). While there was no such reduction in food and water intake of control group animals.

### DEN + CCl4 induced hepatocarcinogenesis coincides with increased lipidosis and fibrosis in hepatocytes

**Figure 2:**
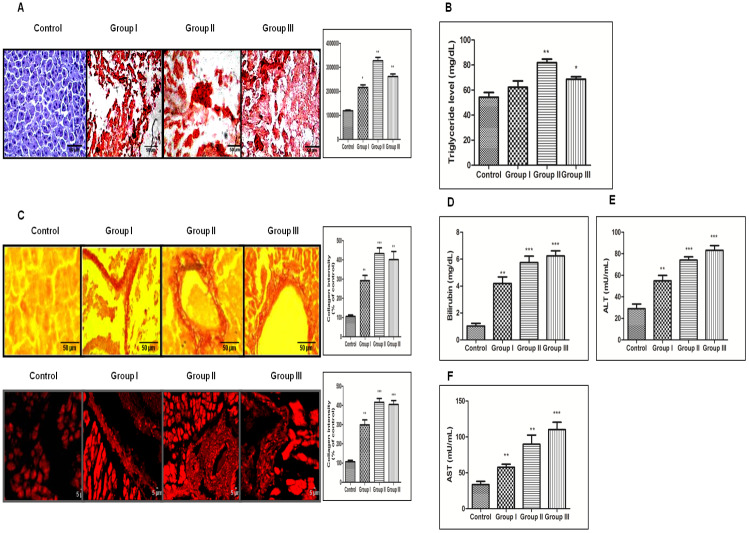
DEN+CCl4 induced hepatocarcinogenesis was associated with increased lipidosis and fibrosis in hepatocytes. **A**. Lipid accumulation by ORO staining. The intensity of positive lipid droplets was calculated by ImageJ software and shown in the graph. **B**. The graph showing triglyserides level in all three groups of DEN + CCl4 treated animals including control one. **C**. Fibrous changes observed in DEN treated groups by Picrosirius red staining. Images were taken with both bright field and fluorescence microscope. Graph showing the collagen intensity as % of control calculated by Image-J software. **D**. Estimation of bilirubin showing constantly increase level in DEN+CCL4 treated animals. **E**. and **F**. Estimation of ALT and AST showing constantly increasing level in DEN+CCL4 treated animals. Data represented are representative of three independent experiments performed in triplicates and expressed as Mean ± SE, *p < 0.05, **p < 0.001, ***p < 0.0001.

After visually observing fat accumulation in and around the liver we next checked the lipid accumulation in rat liver section through Oil Red-O (ORO) staining. As demonstrated in Figure 2A there was strong positive ORO-staining in all three groups of animals except the control group. The staining intensity was significantly more in the group II animals (Figure 2A). Besides, we performed serological analysis of Triglycerides (TG) level in all three treated groups along with the control. Our results complemented the ORO staining read out as the TG level was more in treated groups with enhanced level in group II (Figure 2B).

We also checked the fibrous deposition in DEN + CCl4 treated animals through picro-sirius red staining. The histological evaluation of liver fibrosis was done with the observation of very dark orange stained collagen fiber deposition around enlarged central vein and periportal areas in group II and group III animals (Figure 2C upper-panel). The liver-sections were also observed under fluorescence microscopy and it revealed the similar pattern (Figure 2C lower-panel). These findings were simultaneously associated with increased levels of bilirubin, Alanine aminotransferase (ALT) and Aspartate aminotransferase (AST) enzymes in blood-serum of treated animals (Figure 2D-2F). Furthermore, in order to visualize the histological pattern of treated animals and control animals we performed hematoxylin and eosin (H and E) staining of liver sections of all animals (Supplementary Figure 1).

### Strong positive correlation between the expression of SHH and lipogenic molecules in DEN + CCl4 induced hepatocarcinogenesis model

**Figure 3:**
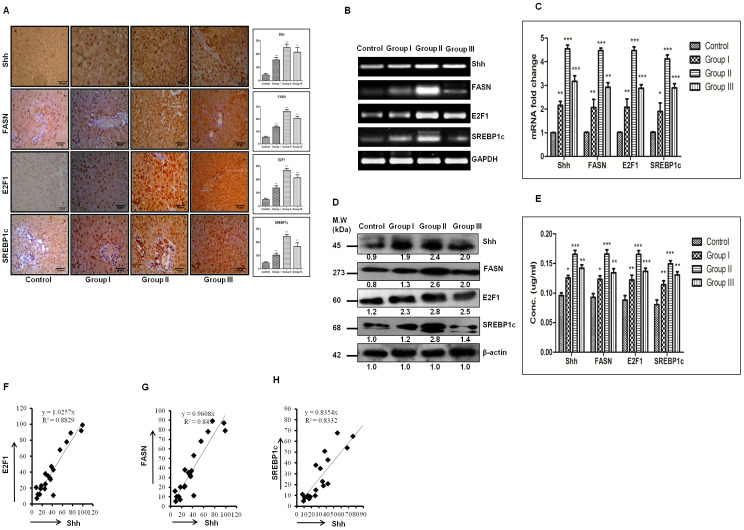
Strong positive correlation between the expression of SHH and lipogenic-molecules in DEN + CCl4 induced hepatocarcinogenesis model. **A**. IHC staining of SHH, E2F1, FASN and SREBP1c in rat liver tissue sections of different stages of hepatocarcinogenesis. The corresponding IHC staining intensity graph is shown in side panel of figures. Y axis shows the labeling index (%) visual score in each intensity graph. **B**. Increasing mRNA expression by RT-PCR method. **C**. Fold increase in relative mRNA expression, calculated with respect to GAPDH for SHH, E2F1, FASN and SREBP1c in control and treated groups. **D**. Expressions of protein was determined by Western blot and densitometric analysis with respect to loading control b-actin. **E**. Quantification of SHH, FASN, E2F1 and SREBP1c by ELISA in rat tissue lysates of different groups of HCC. **F**.–**H**. Graph showing strong positive correlation between SHH and FASN, E2F1 and SREBP1c across all stages of hepatocarcinogenesis. Data represented are representative of three independent experiments performed in triplicates and expressed as Mean ± SE, *p < 0.05, **p < 0.001, ***p < 0.0001.

Activated Hedgehog signaling is known to be associated with fibrogenic response in liver [19] and is also known to control lipogenesis in various human malignancies [20]. This prompted us to investigate the expression of SHH and lipogenic molecules in DEN + CCl4 treated rat liver-sections. As demonstrated by Figure 3A, we can see increased staining of SHH in treated Wistar rats. The number of positively stained cells and staining intensity were more in group II animals (Figure 3A). The expression levels of lipogenic molecules FASN and SREBP1c were increased in treated animals. We also checked E2F1 expression in the same. The mRNA and protein expression level for all three molecules was highest in group II animals. The expression levels of lipogenic molecules, E2F1, and SHH were also checked at mRNA level by RT-PCR and quantitative RT-PCR (Figure 3B–3C) and at protein level by western-blot and ELISA (Figure 3D-3E). We also found a strong positive correlation between SHH and all the molecules analyzed (Figure 3F-3H).

### Enhanced E2F1 was associated with increased expression of adiponectin in DEN + CCl4 model of hepatocarcinogenesis

**Figure 4:**
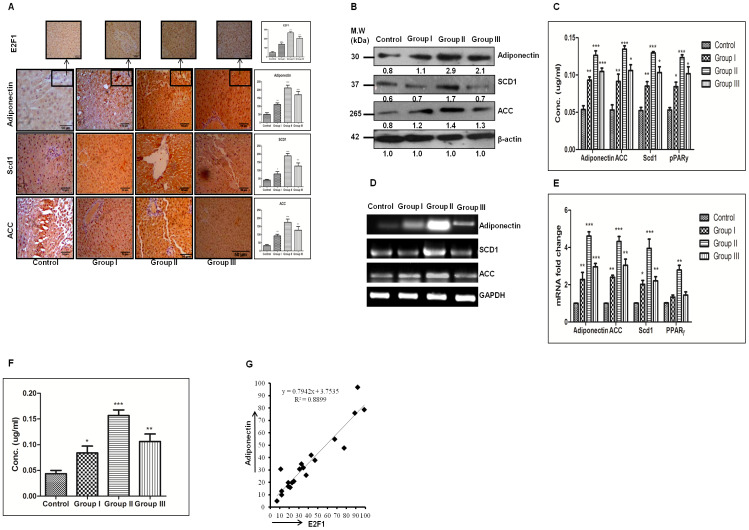
Enhanced E2F1 is associated with increased expression of adiponectin in DEN+CCl4 model of hepatocarcinogenesis. **A**. IHC staining of Adiponectin, SCD1 and ACC in DEN treated rat liver tissue sections of different stages of hepatocarcinogenesis. IHC images of E2F1 staining of the similar section is shown as the top projection. The corresponding IHC staining intensity graph is shown in side panel of figures. Y axis shows the labeling index (%) visual score in each intensity graph. **B**. The Western blot and densitometric analysis with respect to loading control b-actin. **C**. Quantification of lipogenic molecules by ELISA. **D**. Increasing mRNA expression level of lipogenic molecules of control and treated groups. **E**. The figure represents fold increase in relative mRNA expression, calculated with respect of GAPDH. **F**. Quantification of Adiponectin in rat blood serum samples by ELISA. **G**. Graph showing strong positive correlation between E2F1 and Adiponectin across all stages of hepatocarcinogenesis. Data represented are representative of three independent experiments performed in triplicates and expressed as Mean ± SE, *p < 0.05, **p < 0.001, ***p < 0.0001.

E2F1 is one of the well known transcription factors involved in the regulation of lipogenesis [19] and adipogenesis [20]. After we observed increased expression of E2F1 and lipogenic molecules in treated animals, we next checked the expression of adiponectin. As demonstrated by Figure 4A, the E2F1 staining intensity was more in group II animals (Figure 4A Inset) and simultaneously the adiponectin staining intensity was significant in all three groups of treated animals as compared to control (Figure 4A). This increased E2F1 and adiponectin expression was also associated with increased expression of lipogenic molecules acetyl-CoA-carboxylase (ACC) and stearoyl-CoA desaturase (SCD1) (Figure 4A). We further checked the expression levels of these molecules by western blot, ELISA, RT-PCR, and qRT-PCR which show the similar expression pattern as we observed in immunohistochemistry (IHC) staining with maximum expression in group II animals for all molecules (Figure 4B-4E). As adiponectin is a hormone and is secreted in blood flow, so we confirmed its presence in blood-serum through ELISA. Our results showed significant level of adiponectin in blood-serum of all animals and the expression was maximum in group II (Figure 4F). We also demonstrated a strong positive correlation between E2F1 and adiponectin expression in all three groups of animals (Figure 4G). Additionally, we also observed enhanced expression of pAKT in all three groups of treated animals (Supplementary Figure 2).

### Enhanced expression of adiponectin and lipogenic molecules in higher stage hepatocellular carcinoma patients’ biospecimens

**Figure 5:**
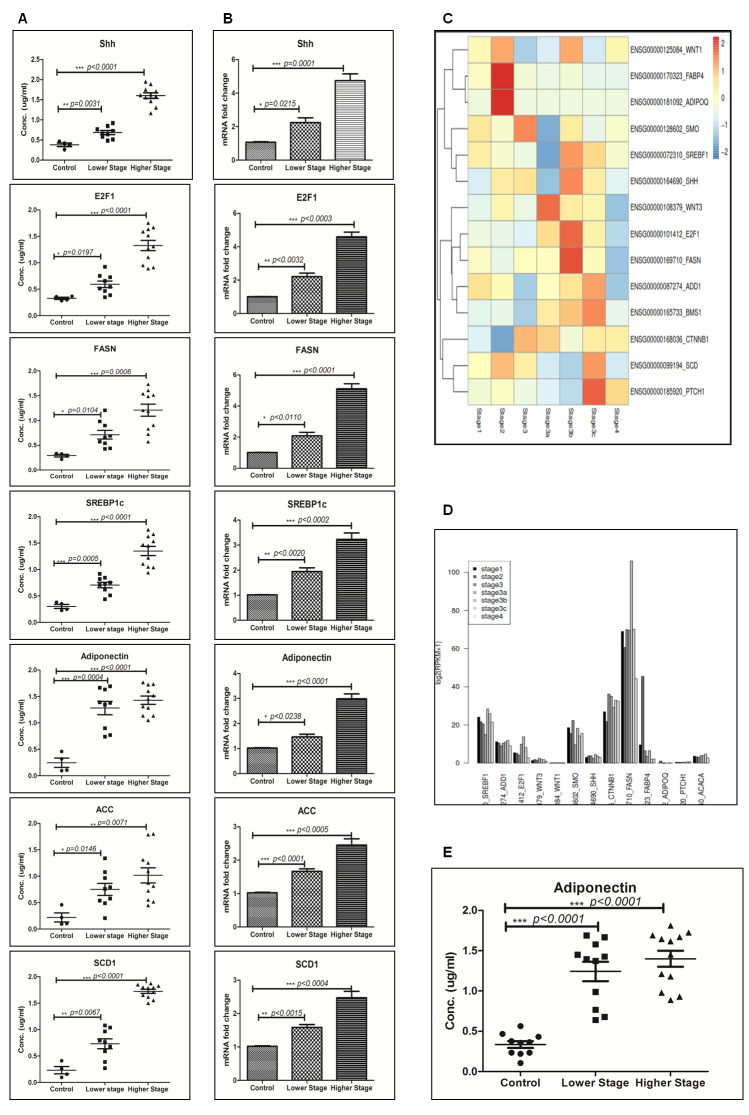
Enhanced expression of adiponectin and lipogenic-molecules in higher-stage hepatocellular carcinoma patients’ biospecimens. **A**. Quantification of lipogenic molecules in hepatocellular carcinoma patients’ samples of different hepatocellular carcinoma stages by indirect ELISA. **B**. The figure represents fold increase in relative mRNA expression, calculated with respect of GAPDH in hepatocellular carcinoma patients’ samples of different hepatocellular carcinoma stages. **C**. The heat map of RNASeq data showing expression level of different SHH and lipogenesis genes with the hepatocellular carcinoma stages/grades. **D**. Figure showing log scale of different SHH and lipogenesis genes with the hepatocellular carcinoma stages/grades. **E**. Quantification of Adiponectin in the blood serum of hepatocellular carcinoma patients of different stages by ELISA. Data represented are representative of three independent experiments performed in triplicates and expressed as Mean ± SE, *p < 0.05, **p < 0.001, ***p < 0.0001.

The translatability of *in vitro* or *in vivo* research findings is incomplete without integration of clinical research findings. So we next performed expression analysis of adiponectin and lipogenic molecules in clinical biospecimen from hepatocellular carcinoma patients’ of different stages. Our results demonstrated expression of adiponectin and lipogenic molecules in lower and higher-stage patients’ biospecimen as compared to control individuals at protein (Figure 5A) and mRNA (Figure 5B) level. The expression level was prominently more in higher-stage patients. Nonetheless, we also performed the expression profile analysis of these molecules in hepatocellular carcinoma patients of different stages available at The Cancer Genome Atlas (TCGA)-database. Our analysis made it clear that there is significantly higher level of expression of lipogenic molecules FASN, SREBP1c, and SCD1 in higher-stage patients as demonstrated in the heatmap (Figure 5C). The heatmap also showed that the expression level of E2F1, SHH, Smo, and Ptch1 were as well more in higher-stage patients (Figure 5C). The expression levels were also checked at Log-scale for a more quantitative analysis. The graph showed very high value of FASN expression followed by SREBP1c and E2F1 (Figure 5D). Besides, our results confirmed significant level of adiponectin in blood-serum of hepatocellular carcinoma patients and the expression was significantly more in higher-stage patients (Figure 5E).

### Pharmacological inhibition of SHH was coupled with decreased expression of adiponectin and lipogenic-molecules in hepatocellular carcinoma cell-line

**Figure 6:**
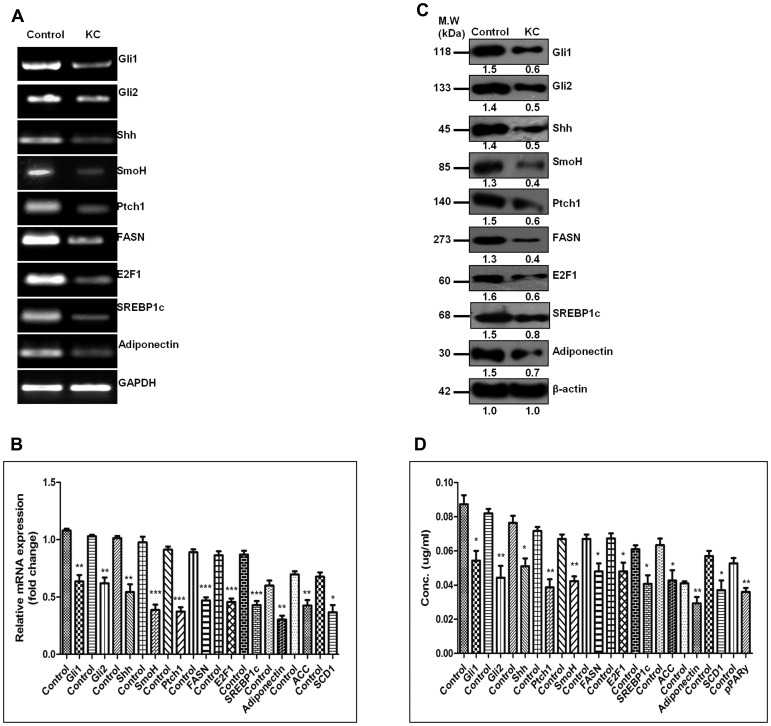
Pharmacologic inhibition of SHH was coupled with decreased expression of adiponectin and lipogenic-molecules in hepatocellular carcinoma cell-line. **A.–B**.The decreasing mRNA expression level of SHH and lipogenic molecules in Hep3B cells after KC treatment with respect of GAPDH. **C**. The Western blot and densitometric analysis of Hh and lipogenic molecules with respect to b-actin in KC treated and untreated cells. **D**. Expression analysis of Hh and lipogenic molecules in KC treated and untreated cells by ELISA method. Data represented are representative of three independent experiments performed in triplicates and expressed as Mean ± SE, *p < 0.05, **p < 0.001, ***p < 0.0001.

**Figure 7:**
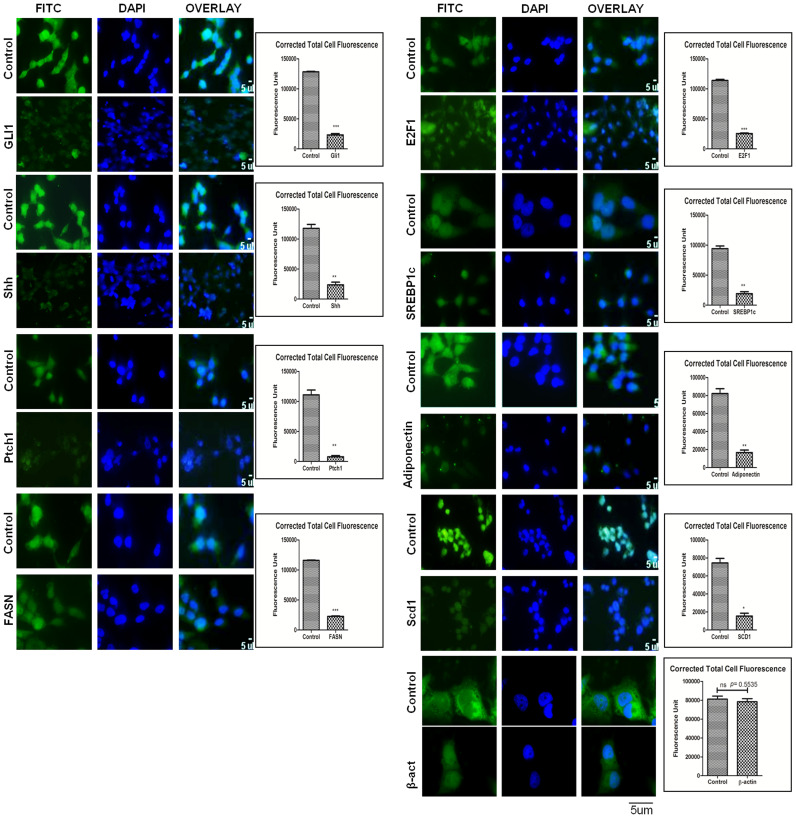
Figure 7: Expression level of Hh and lipogenic molecules in hepatocellular carcinoma cell-line in the presence of KC. Immunocytochemistry staining of Hh and lipogenic molecules in Hep3B cells after KC treatment. Corrected Total Cell Fluorescence showing significantly decrease expression. Data represented are representative of three independent experiments performed in triplicates and expressed as Mean ± SE, *p < 0.05, **p < 0.001, ***p < 0.0001.

To explore the role of SHH in lipogenesis and adipogenesis we inhibited SHH pathway in a hepatocellular carcinoma cell-line Hep3B. We utilized KAAD-Cyclopamine (KC), which is a potent pharmacological inhibitor of SHH signaling pathway and target Smo receptor [21]. Hep3B cells were treated with 2.5 μM KC for 48 h, then the expression levels of adiponectin and lipogenic molecules were checked. The results demonstrated that the expression level of SHH pathway molecules (SHH, GLI1, GLI2, SmoH, and Ptch1), lipogenic molecules (FASN, SREBP1c, E2F1, and Adiponectin) significantly decreased after KC-treatment at mRNA (Figure 6A-6B) and at protein level (Figure 6C-6D) as compared to control for each molecule under analysis. These results were also complemented with the immunostaining intensity after KC-treatment (Figure 7). The immuno-score analysis of each molecule showed significant decrease in the expression level after KC-treatment in comparison to control.

### Pharmacological inhibition of SHH signaling pathway was associated with decreased tumorigenicity of Hep3B cell-line in soft-agarose assay

**Figure 8:**
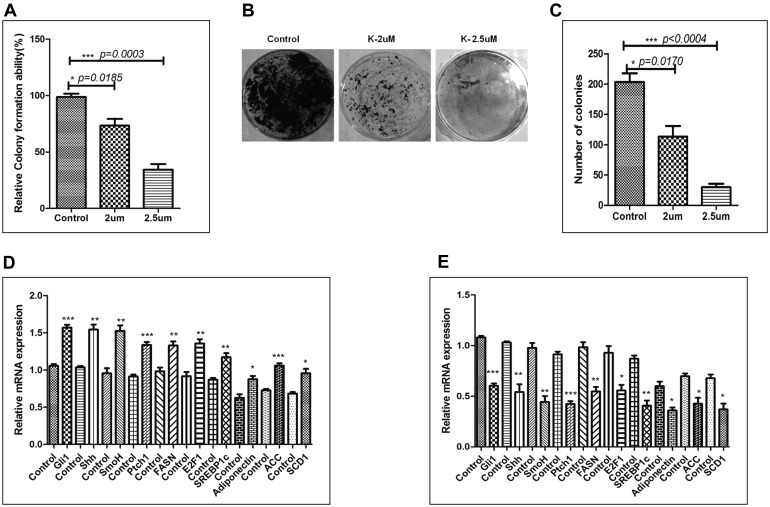
Pharmacological inhibition of SHH signaling pathway is associated with decreased tumorigenicity of Hep3B cell-line in soft-agarose assay. **A**. Relative colony formation ability (%) was measured and shown in the graph. **B**.–**C**. Number of colonies were counted and plotted in the graph. (**D**–**E**) Relative mRNA expression of SHH and lipogenic molecules with respect of GAPDH after 48 hrs treatment with Pur and Pur + KC along with their respective controls. Data represented are representative of three independent experiments performed in triplicates and expressed as Mean ± SE, *p < 0.05, **p < 0.001, ***p < 0.0001.

The soft-agarose colony formation assay is one of the most precise assays to test the anchorage independent growth of cell-lines *in vitro*. To test the impact of KC-treatment on tumorigenicity of Hep3B cell-line *in vitro* we performed soft-agarose colony formation assay. As demonstrated by Figure 8A there is significant decrease in the tumorigenicity of Hep3B cells after KC-treatment. The relative colony forming ability was significantly less in KC-treated group as compared to control (Figure 8A). The number of colonies was also less in treated cells as demonstrated pictorially and graphically in Figure 8B-8C. The Figure 8B shows pictures of colonies taken after completion of the soft agarose colony formation assay. On the other hand Figure 8C shows number of colonies counted in control and treated samples. Additionally, we exogenously stimulated SHH pathway to see the impact of KC-treatment in Hep3B cells. Pumorphamine (Pur) is a known activator of Hedgehog pathway which activates Hh pathway by directly binding to Smo receptor [22]. We activated Hh pathway above basal level by treating Hep3B cells with Pur (2 μM for 48 hrs). Cells were then treated with KC (2.5 μM for 48 hrs) afterward qRT-PCR analysis was done to quantitate the Hh pathway and lipogenic molecules. The results confirmed the activation of Hh and downstream molecules after Pur-treatment (Figure 8D) which get significantly decreased when cells were further treated with KC (Figure 8E).

## DISCUSSION

Hepatocellular carcinoma is one of the cancers which show strong association with fat contents in the body. The multistep process of hepatocellular carcinoma development has been intensely investigated in several pre-clinical models including Wistar rats [18]. The development of hepatocellular carcinoma is associated with deregulation of several growth and proliferative signaling pathways. Among them the SHH signaling pathway and their role in process of hepatocarcinogenesis is well documented [23]. Although, the SHH pathway is activated in hepatocellular carcinoma but its role in regulating lipogenesis during the process of hepatocarcinogenesis is not documented. Though the role of Indian Hedgehog signaling pathway in high fat diet induced hepatocarcinogenesis is documented earlier [24], but the study did not consider SHH pathway and lipogenic molecules. In the present study, we explored the role of SHH and associated lipogenic sketch during the process of hepatocarcinogenesis utilizing both experimental and correlation approaches.

Several studies have been done to investigate the molecular changes associated with DEN induced hepatocarcinogenesis [25], but none of the studies directly investigated DEN induced fatty metamorphoses during hepatocarcinogenesis. There are few studies which investigated the impact of high fat diet on DEN induced hepatocarcinogenesis, where high fat diet worked as a promoter [26]. In our DEN + CCl4 induced hepatocarcinogenesis study we sacrificed animals at three different time points to investigate the correlation between SHH and lipogenic molecules at different stages of hepatocarcinogenesis. Our results demonstrated that after DEN + CCl4 treatment there was significantly higher amount of visible visceral fat deposition in and around the liver of animals (Figure 1A). Additionally, all three groups of treated animals showed incidence of multi-nodular tumors (Figure 1B). Our results revealed that the relative liver-weight was increased in all treated animals as compared to control across the experimental period irrespective of hepatocarcinogenesis stage (Figure 1C). This result demonstrates that active proliferation is going on after DEN + CCl4 treatment which was also confirmed in our previous finding [18]. A decrease in appetite was observed in all treated groups as compared to control in chronological order (Figure 1D) and it was simultaneously associated with decreased water intake (Figure 1D).

As we saw increased fat in and around the liver of treated animals, so we checked the accumulation of lipids in the liver sections with Oil Red O (ORO) staining. The result clearly demonstrates highly significant lipid accumulation in rat hepatocytes of DEN + CCl4 treated animals with normal diet feeding and the staining intensity was more in group II (Figure 2A). We also performed serological analysis of total triglyceride (TG) content in blood serum of animals in all four groups. The result complemented our ORO staining results with higher triglyceride content in DEN + CCl4 treated animals and maximum triglyceride content was in group II (Figure 2B). Our next result showed positive staining for picro-sirius red in treated animals. Picro-sirius red staining is known to highlight the collagenous component and this dye is the most recommended as it is known to color all collagens intensely and selectively [27]. Our results revealed dark orange stained large collagen fibers around the enlarged central vein and portal triads were easily visible under microscope in all treated groups (Figure 2C upper panel). A progression in liver fibrosis was observed in group II and III animals as compared to group I and there was visible formation of fibrous septa around the enlarged central vein and portal triads. The liver sections were also observed under fluorescence microscopy and it revealed the similar pattern of picro-sirius red staining across the three groups (Figure 2C lower panel). We further checked the serum level of bilirubin, AST (Aspartate aminotransferase) and ALT (Alanine aminotransferase) enzymes to assess the extent of liver damage after DEN + CCl4 treatment. A progressively increased value of AST, ALT enzymes along with bilirubin indicated severe liver damage across all three groups (Figure 2D-2F). We also performed histological analysis for all four experimental animal groups and the results revealed disorganized hepatic architecture in all DEN + CCl4 treated groups (Supplementary Figure 1).

After we observed increased lipogenesis and fibrosis in our DEN + CCl4 induced hepatocarcinogenesis model, we next sought to find a common molecular pathway which is involved in both lipogenesis and fibrosis. Our previous work demonstrated activation of SHH signaling pathway in DEN + CCl4 induced hepatocarcinogenesis animal model [18]. Furthermore, it has been reported that activated hedgehog pathway play an important role in fibrosis [19] and lipogenesis [20] in various human malignancies. So we subsequently checked the expression of SHH and its downstream molecules which might be involved in lipogenesis. The expression of SHH, E2F1, FASN and SREBP1c were checked in all groups. As demonstrated by our results the expression level of these molecules were increased in group I, II, and III in comparison to control and the staining intensity was significantly more in group II animals (Figure 3A). This finding was also complemented with other techniques like RT-PCR, qRT-PCR, western blot, and ELISA (Figure 3B–3E). E2F1 transcription factor is known to be directly regulated by retinoblastoma (RB)-pathway [28], one of the most frequently inactivated pathways in HCC [29]. The tumor suppressor protein retinoblastoma and its family members are known to inhibit transcription of E2F responsive genes which are involved in cell cycle progression [28]. Diseases like diabetes and obesity is associated with inhibition of glucose oxidation which in-turn is associated with increased lipogenesis following retinoblastoma inactivation and consequent E2F1 activation [30]. Bhatia et al. (2011) demonstrated that in SHH driven medulloblastoma there is inactivation of retinoblastoma which in turn causes activation of E2F1 and consequently enhanced expression of lipogenic-molecules [20]. Our results corroborated these findings in hepatocellular carcinoma model and notably, we also found a strong positive correlation between the SHH expression and the expression of E2F1, FASN, and SREBP1c (Figure 3F-3H).

E2F1 transcription factor is also involved in regulation of adipogenesis [31], so we further demonstrated the expression of adiponectin in all groups. We found significantly increased level of adiponectin in all the treated animals and it showed maximum expression level distinctly in group II animals at mRNA and protein level (Figure 4A-4E). We also checked expression of other signaling molecules involved in lipogenesis like ACC, SCD1, and PPAR-γ (Figure 4B-4E) and the expression pattern of all these molecules coincide with that of adiponectin. Adiponectin is an adipokine so we also checked its expression in blood-serum and demonstrated enhanced expression of adiponectin in group II animals (Figure 4F). Simultaneously, there was strong positive correlation between adiponectin and E2F1 expression (Figure 4G). Several investigators have demonstrated the inverse relation between adiponectin expression and incidence of various cancer types [32, 33]. But in case of hepatocellular carcinoma the data regarding adiponectin expression and hepatocellular carcinoma clinicopathology is complex. Some studies with pre-clinical models have shown that adiponectin expression is associated with reduced tumor growth, proliferation, and metastasis [34, 35]. Furthermore, there are quite a bit number of studies which illustrated the supporting effect of increased adiponectin on hepatocarcinogenesis [36-38]. Siegel et al. demonstrated the association between high serum adiponectin level and worsened overall survival in hepatocellular carcinoma patients in a prospective cohort study [38]. In another study, Wang et al. demonstrated poor survival of hepatocellular carcinoma patients with high adiponectin levels was associated at least in part through increased activation of AKT pathway [37]. In similar way our results demonstrated the increased expression of adiponectin particularly in group II animals which was also associated with higher expression level of pAKT in the same group (Supplementary Figure 2).

We also checked the expression of SHH and lipogenic molecules in hepatocellular carcinoma patients’ FNAC (Fine Needle Aspiration Cytology) samples. Our results clearly demonstrated higher expression level of these molecules in higher-stage hepatocellular carcinoma patients’ (Figure 5A-5B) at protein and mRNA level. The results demonstrated enhanced expression of SHH, E2F1, adiponectin, and lipogenic molecules in higher-stage patients as compared to lower-stage patients (Figure 5A-5B). At the same time the expression level of lipogenic molecules like, FASN, SREBP1c, ACC, and SCD1 were more in higher stage patients (Figure 5A-5B). The expression of adiponectin in patients’ blood-serum was also more in higher-stage patients as compared to the lower-stage (Figure 5E). Furthermore, the heatmap from TCGA data-analysis shows more expression of SHH, E2F1, and lipogenic molecules like FASN and SREBP1c in higher-stage patients’ (Stage3a and above) (Figure 5C). This result was complemented with a more quantitative analysis of these molecules by plotting RPKM values on log scale (Figure 5D). Bar-plot of absolute expression values revealed the superlatively high expression of FASN in higher-stage (stage3b) followed by SREBP1c. The expression of SHH, Smo, and E2F1 were also significantly high (Figure 5D). However, the heatmap showed increased expression of adiponectin in lower-stage patients (Stage 2). The reason for this discrepancy from our cross-sectional observational study might be possibly because of variability in the disease etiology and small sample size. Though, our results are well supported with few published reports underscoring the role of high adiponectin in exacerbating hepatocellular carcinoma clinicopathology [37, 38].

Furthermore, our results demonstrated that inhibition of SHH pathway coincides with reduced expression of adiponectin and lipogenic molecules in HCC cell-line. Adiponectin is known to be differentially expressed by various human cell-lines. Bergner et al., (2018) demonstrated the expression level of adiponectin by various human cell-lines including the liver adenocarcinoma cell line SK-HEP-1 and some other cancer cell-lines [39]. Although the expression level of adiponectin in HCC cell-line Hep3B has not been reported earlier; but, reports suggest adiponectin expression by liver carcinoma cell-lines and also by other carcinoma cell-lines. We are the first to report the expression level of adiponectin by Hep3B cells (Figure 6A). The effectiveness of KC-treatment was also observable with the reduced expression of Gli1 and Ptch1, the direct transcriptional target of SHH pathway. This was simultaneously associated with decreased expression of E2F1, FASN, SREBP1c, adiponectin (Figure 6A). Other lipogenic molecules like ACC and SCD1 also reduced significantly. These results were clearly evident at both mRNA and protein level (Figure 6B-6D). The similar pattern of reduced expression was also observable in Hep3B cells after KC treatment through immunocytochemistry technique (Figure 7) supporting the role of SHH pathway in regulation of lipogenesis. The tumorigenic potential of Hep3B cells were checked after KC treatment through soft-agarose assay. It is evident through the results that the relative colony forming ability of Hep3B cells was significantly reduced after KC treatment (Figure 8A). Moreover, there was a considerable decrease in total number of colonies after KC treatment (Figure 8B-8C) confirming the inhibition of SHH pathway as an anti-hepatocarcinogenic strategy via. E2F1-axis. We elaborated our finding by exogenous activation of SHH pathway through Pur-treatment followed by KC-treatment. We investigated the activity of SHH pathway and its control over lipogenesis by using Pur, a pharmacological activator of SHH pathway. The qRT-PCR results demonstrated significant increase in the expression of Patch1 and Gli1 confirming the functional responsiveness of SHH pathway after Pur-treatment. Additionally, the expression of FASN, E2F1 and adiponectin also increased significantly (Figure 8D). Furthermore, we demonstrated that the exogenous activation of SHH pathway also responded to the SHH inhibitor treatment. Following KC-treatment of Pur-treated cells demonstrated significant reduction in almost all functional components of SHH pathway and lipogenic molecules (Figure 8E), implicating the role of SHH pathway in lipogenesis.

## MATERIALS AND METHODS

### Cell culture and treatments

Hep3B hepatocellular carcinoma cell-line was obtained from National Centre for Cell Science, Pune, India. The Hep3B cell-line was authenticated with short tandem repeat (STR) profiling in the parent organization before procuring. The profiling was done in March 2018. Once we obtained the cell-line in Disease Biology Lab, KIIT School of Biotechnology, we passaged cells for two weeks before cryo-preserving the cells. Thereafter the cells were regularly checked for their morphology and growth-curve analysis, and were never passaged for more than 30 days in a stretch. For experiments, cells were maintained in DMEM, supplemented with penicillin-streptomycin (100 U/ml) and 10% fetal bovine serum at 37° C in 5% CO_2_ – 95% air atmosphere in a humidified incubator. Cells were sub cultured every 5 to 6 days.

### Soft-agar colony formation assay

For anchorage-independent growth assay, in 6 well plates, base-agar was made by 0.75% agarose in culture media. Top-agarose overlay was made fresh by mixing 0.5% molten agarose with 2 × culture media containing 2500 cells/well and gently overlaid over the base-agar. The plate was kept for solidification at 37° C thereafter the cells were overlaid with DMEM supplemented with 10% FBS. The plates were incubated for 21 days and the medium was replaced twice a week. The colonies were stained with crystal-violet and counted visually. Finally, the stain was dissolved in 10% glacial acetic acid and the absorbance of the released dye was measured at 590 nm.

### *In vivo* experiments and hepatocarcinogenesis model

*In vivo* studies were performed with male Albino Wistar rats of 120–150g body weight which were procured from the laboratory animal facility of KIIT School of Biotechnology. They were housed in standard temperature/ humidity conditions and environment (12 hr light/dark cycle) [18]. All animals were provided standard pellet diet and water ad libitum. All the experimental protocols were approved by the Institutional Animal Ethics Committee (IAEC, KIIT School of Biotechnology, Bhubaneswar, India) Approval number- KSBT/IAEC/2017/MEET-1/A3).

The rats were randomly and evenly allocated into six groups, six rats in each group. All animals were acclimatized for two weeks before starting the experiment. The DEN + CCl4 model of rat hepatocarcinogenesis was followed in our experiment [18]. Rats were randomly and evenly allocated into Control, Group I, Group II, and Group III were assigned as initiation stage, promotion stage and progression stage animals. N-Nitrosodiethylamine (DEN, Sigma Aldrich) was administered in group I, II, and III as 100 mg/kg body weight per week for three consecutive weeks. After 1 week of recovery period, the promoting reagent CCl4 (Carbon tetrachloride) was given 2 ml/kg body weight of rats weekly twice for consecutive 8 weeks. At the completion of the 8 weeks treatments, and after latency period of two weeks we observed altered hepatic foci or morphologically recognizable lesions in DEN treated animals. The rats were given same treatment and sacrificed under anesthesia at different time points for each group which were separated by the interval of one week and before sacrifice the total food-water intake and liver-body weights were recorded.

### Quantitative real-time PCR

Total RNA was extracted with TRIzol reagent in accordance with the manufacturer’s instructions. Reverse transcription was performed in total volume of 20 μl using 2 μg of total RNA by Verso c-DNA synthesis kit. To quantify the changes in m-RNA level, reverse transcription PCR and RT-qPCR were performed on the CFX ConnectTM Real-Time PCR Detection System (Life-Technology, BIORAD). RT-PCR was performed by using Go Taq Green Master Mix. PCR primers were designed based on human mRNA sequence. PCR products were separated by electrophoresis in 1% agarose gel, visualized by 0.5 μg/ml ethidium bromide staining for 40 mnts. The gel image and intensity of each band was measured by GEL-DOC Image software (BIO-RAD). q-RTPCR was performed by using SsoFast EvaGreen Supermix (Cat: 1725203, BIORAD) with the following cycling conditions: 95° C for 5 min, followed by 32 cycles of 95° C for 15 sec and 60° C for 25 sec. Glyceraldehyde 3- phosphate dehydrogenase (GAPDH) was used as housekeeping gene. The primer details and cycles are described in the Supplementary Table 1.

### Immunocytochemistry

Hep3B cells were grown on glass coverslips and treated with KAAD-Cyclopamine (KC, 2.5 μM) for 48 hrs. Cells were fixed with 3.7% paraformaldehyde, made permeable with 0.1% Triton-X-100 and blocked with 1% BSA. Cells were probed with primary antibody (1:1000 or 1:2000) and FITC fluorophore tagged secondary antibody (1:3000). Then cells were counterstained with DAPI, washed, mounted and viewed under fluorescence microscope Olympus (BX 61). Capturing of the images and cell fluorescence was measured using Image Pro Express (40X, scale bar-5 μm) and Image-J software. Corrected Total Cell Fluorescence (CTCF) was calculated using the formula.

CTCF = Integrated Density – (Area of selected cell × Mean fluorescence of background readings) (“Measuring cell fluorescence using ImageJ — The Open Lab Book v1.0,” n. d.).

### Biochemical analysis of serum

The level of clinical biochemical parameters such as alanine aminotransferase (ALT), aspartate aminotransferase (AST) and bilirubin were evaluated to determine the enzymatic activities of the liver of control and experimental groups by using commercially available kits (Sigma-Aldrich) according to the manufacturer’s instructions.

### Protein extraction and Western blotting

To check the expression of proteins, western blot analysis was carried out by using conventional homogenization method using liquid nitrogen. The pellet was resuspended in RIPA lysis buffer. Total of 50 μg of protein lysates were separated on 10% SDS-PAGE. The gel was then subjected to electro-blotting on PVDF membrane. The membrane was blocked with 5% skimmed milk in TBST for 2 hrs at RT and then probed with the primary antibody (1:1000 or 1:2000) for 3 hrs at RT or at 4° C overnight. This was followed by washing the membrane with TBST and probed with secondary antibody (1:2500) for 2 hrs at RT. The blots were visualized by enhanced chemiluminescence using X-ray film.

### ELISA

The detection of serum samples collected from rat liver-tissues, whole cell lysates and human patients’ Fine needle aspiration cytology (FNAC) samples was done by Indirect ELISA method according to our established laboratory protocol. Briefly, the protein antigen (1 μg/ml conc.) was mixed with coating buffer (0.05 M) and coated onto 96 well microplate. The plate was then incubated overnight at 4° C followed by washing with 1X PBST then blocking with 1% BSA at room temperature for 2 hrs. Primary anitibodies (1:2000 dilution) were added into each well and incubated 2 hrs at room temperature followed by washing thrice with 1X PBST. The wells were then incubated with secondary HRP linked antibody (1:2500 and 1:5000 dilutions according to the primary antibody) for 45 mints at room temperature. After washing thrice with 1X PBST, 2,2’-azino-bis (3-ethylbenzothiazoline-6-sulphonic acid) or ABTS substrate solution was added and absorbance was read at 410 nm using ELISA microtitreplate reader (EPOCH).

### Immunohistochemistry

Immunohistochemistry analysis was done for tissue sections taken over poly-L-lysine coated slides. The tissue sections were taken from formalin fixed, paraffin embedded blocks. Sections were cut 4 μm thick. Immunostaining was done by the use of Rabbit Specific HRP/DAB (ABC-IHC) Kit. The experiment was done according to the manufacturer’s protocol. Imaging of the sections was done using Leica (DM 2000) bright-field microscopy (40X, scale bar-50 μm) and the intensity score was calculated by Immuno-Ratio Analysis software.

### Oil Red-O (ORO) and picro-sirius red staining

A) ORO-staining was done for paraffin-embedded tissue sections. The sections were deparaffinized and fixed in 3.7% paraformaldehyde followed by washing with 60% isopropanol. The sections were stained with filtered ORO-stain, rinsed with dH^2^O and again stained with hematoxylin followed by washing with 1× TBS thrice.

B) For Picro-sirius red staining, the tissue section were deparaffinized and hydrated in xylene followed by alcohol and dH^2^O. Then sections were stained with adequate picro-sirius red solution, incubated and washed with two changes of acidified water. Most of the water were removed by vigorous shaking and quickly dehydrate in three changes of 100% alcohol then cleared in xylene.

Finally the sections (A&B) were mounted with mounting media and visualized under Leica (DM 2000) bright-field (40X, scale bar-50 μm) and fluorescence microscope Olympus BX-61(40X, scale bar-5 μm).

### Hepatocellular carcinoma patients’ sample and TCGA data analysis

FNAC samples were obtained from 4 control and 20 hepatocellular carcinoma patients. The blood samples were obtained from 10 control and 23 hepatocellular carcinoma patients belonging to different stages. These biospecimens were obtained from department of Gastroenterology and Hepatobiliary Sciences, IMS and SUM Hospital, Bhubaneswar, India. The protocol was approved by Institutional Ethical Committee (Approval no. DMR/IMS-SM/SOA/170029) and written consent was taken from the patients. Patients belonging to stage 2 and 3 were categorized as lower stage and patients belonging to stage3a and above were categorized as higher stage patients as per BCLC (Bercelona Clinic Liver Cancer) staging system. The information regarding patients age and stage are given in Supplementary Table 2.

The Cancer Genome Atlas (TCGA) data-base was utilized for accessing hepatocellular acrcinoma patients’ genomic datasets for different stages. The TCGA data-base is proffered with large scale genomic datasets for analysis which allowed us to utilize it for our analysis on extensive co-operation basis. The TCGA LIHC datasets were analyzed consisting of maximum 10 patients in different stages of hepatocellular carcinoma. Not all stages had 10 patients as for some stages maximum available patient data was limited (Stage1: 10, Stage2: 10, Stage3: 6, Stage3a: 9, Stage3b: 8, Stage3c: 10, Stage4: 6).

### Statistical analysis

The data presented is the Mean±SEM of three independent experiments. Changes in gene expressions were analyzed by two-way analysis of variance (ANOVA) and Student’s t-test using Grpah-Pad Prism version5. *p < 0.05, **p < 0.001, ***p < 0.0001 were considered to be significant.

## CONCLUSIONS

Our study presents atypical niche for SHH pathway through its control over lipogenesis in hepatocellular carcinoma animal and cell model. Our data present an insight into the lipogenic properties of DEN + CCl4 induced hepatocarcinogenesis and correlatively demonstrated how SHH pathway operate to arbitrate this response. Correlative studies with biospecimens also indicated the possibility of SHH signaling pathway to control the lipogenesis in HCC patients’ of different stages. Altogether our study implicate the potential of targeting SHH pathway for decreasing lipogenesis and consequent hepatocarcinogenesis via. E2F1 axis.

## SUPPLEMENTARY MATERIAL AND TABLES



## ABBREVIATIONS

HCC: Hepatocellular Carcinoma
DEN: N-Nitrosodiethylamine
CCl4: Carbontetrachloride
SHH: Sonic Hedgehog
FASN: Fatty Acid Synthase
ACC: Acetyl-CoA-carboxylase
SCD1: Stearoyl-CoA desaturase
desaturase: Stearoyl-CoA desaturase
SREBP1: Sterol regulatory element-binding protein1
RB: Retinoblastoma
Hh: Hedgehog
KC: KAAD Cyclopamine
TG: Triglycerides
ALT: Alanine aminotransferase
AST: Aspartate aminotransferase
FNAC: Fine needle aspiration cytology
ORO: Oil Red-O
Pur: Purmorphamine
TCGA: The Cancer Genome Atlas
GAPDH: Glyceraldehyde 3- phosphate dehydrogenase
IHC: Immunohistochemistry
