# Supplementary material for: Fatty changes associated with N-Nitrosodiethylamine (DEN) induced hepatocellular carcinoma: a role of sonic hedgehog signaling pathway
Source: Genes Cancer. 2020;11(1-2):66–82. doi: 10.18632/genesandcancer.203 (PMC7289904; doi:10.18632/genesandcancer.203)
Supplement: Supplementary file 1 [file ganc-11-66-s001.pdf]

Fatty changes associated with N-Nitrosodiethylamine (DEN) induced hepatocellular carcinoma: A role of sonic hedgehog signaling pathway – Tripathy et al

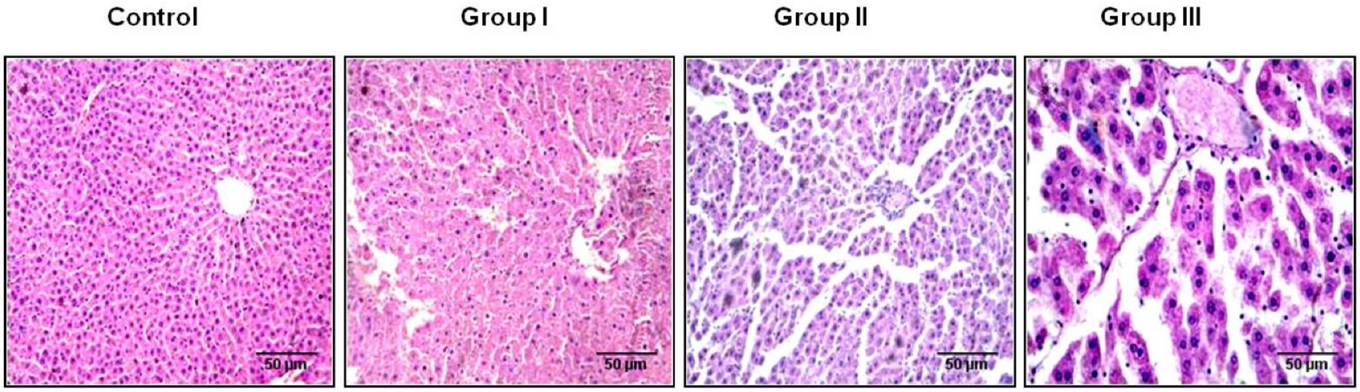

Supplementary Figure 1: Histological pattern of rat liver sections after DEN + CCl4 treatment in all three groups of animals. H&E stained liver sections of control and group I, II, III of DEN + CCl4 treated animals. A routine histopathology was done for the paraffin embedded tissue sections. The pathological analysis of control and treated animals were done by evaluating both macroscopic and microscopic features of liver tissues.

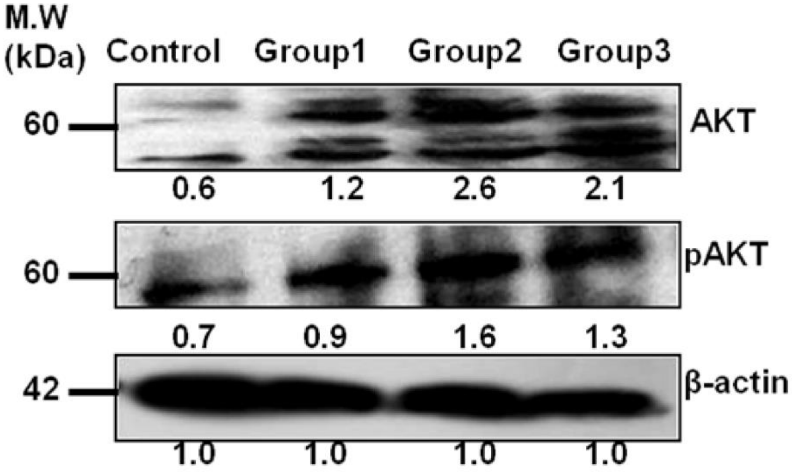

Supplementary Figure 2: Activation of AKT after DEN + CCl4 treatment in all three groups of animals. Expressions of protein was determined by Western blot and densitometric analysis with respect to loading control β-actin. Data represented are representative of three independent experiments performed in triplicates and expressed as Mean ± SE, *$p < 0.05$, **$p < 0.001$, ***$p < 0.0001$.

Supplementary Table 1: Primer set of various genes adopted in RT-PCR

Genes	Sense	Anti-Sense	Annealing temp.	No. of cycles
Gli1	GGGATGATCCCA CATCCTCAGTC	CTGGAGCAGCCC CCCCAGT	56.6° C	34
Gli2	GCAGGTGTATCCC ACGGAAAGCACT G	CTCTCCTCGGCGA GGCTGGTGAGCA T	57.9° C	30
Shh	GATGTCTGCTGCT AGTCCTCG	CACCTCTGAGTCA TCAGCCTG	55.3° C	34
SmoH	GTTCTCCATCAAG AGCAACCAC	CGATTCTTGATCT CACAGTCAGG	55.3° C	32
Ptch1	CAGAGAAGGCTT GTGGCCAC	GCTCAATGACTTC CACCTTCG	56.2° C	32
FASN	AACTCCTTGGCGG AAGAGA	TAGGACCCCGTG GAATGTCA	55° C	32
E2F1	ACTCCTCGCAGAT CGTCATCATCT	GGACGTTGGTGA TGTCATAGATGCG	54° C	30
SREBP1c	ATCGGCGCGGAA GCTGTCGGGGTA GCGTC	GATGTCGTTCAAA ACCGCTGTGTGTC CAGTTC	55.2° C	34
Adiponectin	CGCTTATGTGTAT CGCTCAGG	AGAACTTGCCAG TGCTGTTGTCA	55° C	32
SCD1	CGCTCTTTACCCT TTGCTG	ATAGTCAGTTGCT CGCCTCAC	54.6° C	30
ACC	GGGACTTCATGA ATTTGCTGATTCT CAGTT	GTCATTACCATCT TCATTACCTCAAT CTC	53° C	32
PPARγ	ACCCAATGGTTGC TGATTAC	CGGGAAGGACTT TATGTATGAG	52.5° C	34
GAPDH	ATCTTCCAGGAGC GAGATCCC	CGTTCGGCTCAGG GATGACCT	58° C	30

Supplementary Table 2: Patients details with HCC grade/stage

Sl. No	Age	Gender	Stage/Grade	*Cause*
1	72	Male	Stage-4	CHB, HCC, HTN
2	35	Male	Stage-2	CHB, DCLD, Esophageal varices with active bleeding
3	50	Male	Stage-2	HBV, HCC, Hyperglycemia
4	35	Male	Stage-3c	CHB, GI bleed
5	80	Male	Stage-3	Multi centric HCC, DM, HTN
6	57	Male	Stage-2	CHB, DCLD, Variceal bleed
7	70	Male	Stage-2	CHB, Acute on chronic liver failure
8	63	Female	Stage-3	CLD, 9HCC+UTI+Derrans ed LFT
9	56	Male	Stage-3c	Diagnosis-T2DM, HTN, HBV+V, HCC
10	76	Male	Stage-3	CHB, HCC, HTN
11	51	Female	Stage-3c	CHB, HTN, HCC
12	73	Male	Stage-3a/3b	CHB, HCC
13	52	Male	Stage2	CHB, DCLD, Variceal bleed
14	64	Female	Stage3a/3b	CHB, HCC
15	73	Male	Stage4	CHB, HTN, HCC
16	48	Male	Stage3a/3b	CHB, HCC

17	82	Male	Stage 3a/3b	CHB, HCC, more bleeding
18	55	Male	Stage2	CHB, Acute on chronic liver failure
19	45	Male	Stage3c	Multi centric HCC, DM, HTN
20	62	Male	Stage4	CHB, DCLD, HTN, GI bleed
